# Exploring the prognostic significance of arm-level copy number alterations in triple-negative breast cancer

**DOI:** 10.1038/s41388-024-03051-y

**Published:** 2024-05-14

**Authors:** Samuel Doré, Mariam Ali, Mark Sorin, Sheri A. C. McDowell, Lysanne Desharnais, Valérie Breton, Miranda W. Yu, Azadeh Arabzadeh, Malcolm I. Ryan, Simon Milette, Daniela F. Quail, Logan A. Walsh

**Affiliations:** 1 Rosalind and Morris Goodman Cancer Institute, McGill University, Montreal, QC Canada; 2 Department of Human Genetics, McGill University, Montreal, QC Canada; 3 Department of Physiology, Faculty of Medicine, McGill University, Montreal, QC Canada; 4 Department of Surgery, McGill University Health Center, Montreal, QC Canada; 5 Department of Experimental Medicine, McGill University, Montreal, QC Canada

**Keywords:** Breast cancer, Cancer genomics

## Abstract

Somatic copy number alterations (SCNAs) are prevalent in cancer and play a significant role in both tumorigenesis and therapeutic resistance. While focal SCNAs have been extensively studied, the impact of larger arm-level SCNAs remains poorly understood. Here, we investigated the association between arm-level SCNAs and overall survival in triple-negative breast cancer (TNBC), an aggressive subtype of breast cancer lacking targeted therapies. We identified frequent arm-level SCNAs, including 21q gain and 7p gain, which correlated with poor overall survival in TNBC patients. Further, we identified the expression of specific genes within these SCNAs associated with survival. Notably, we found that the expression of *RIPK4*, a gene located on 21q, exhibited a strong correlation with poor overall survival. In functional assays, we demonstrated that targeting *Ripk4* in a murine lung metastatic TNBC model significantly reduced tumor burden, improved survival, and increased CD4^+^ and CD8^+^ T cell infiltration. RIPK4 enhanced the survival of triple-negative breast cancer cells at secondary sites, thereby facilitating the formation of metastatic lesions. Our findings highlight the significance of arm-level SCNAs in breast cancer progression and identify RIPK4 as a putative driver of TNBC metastasis and immunosuppression.

## Introduction

The Cancer Genome Atlas (TCGA) has enabled the discovery of key genomic changes in over 30 types of tumors [1] and has revolutionized our understanding of cancer prevention, diagnosis, and therapy. Somatic copy number alterations (SCNAs) affect a larger fraction of the genome in cancer than do any other type of genetic alteration [2–4]. SCNAs can be focal (small regions of DNA that are lost or duplicated) or arm-level (loss or duplication of entire chromosome arms). Focal SCNAs are frequent genetic events that can promote tumor initiation, metastasis and resistance to therapy [5], and their discovery has been integral to the identification of many cancer genes. However, little is known about larger arm-level SCNAs, despite occurring 30 times more frequently than expected based on their size [6]. These broad regions of DNA that are gained or lost can include hundreds of genes, many of which are likely bona fide cancer genes and may promote malignant phenotypes [4]. However, pinpointing important genes within large arm-level SCNAs is difficult and remains a hurdle in cancer research with untapped therapeutic value.

We previously developed a comprehensive strategy to identify important genes within arm-level SCNAs in cancer [7]. This approach considers the frequency of an arm-level event, the significance, and the correlation with survival outcome as a multi-tier metric to develop a composite score for each event. We have now extended these analyses to breast cancer (BC), given the high frequency of SCNAs in this disease [8–10]. Triple-negative breast cancer (TNBC) is an aggressive BC subtype accounting for 10–20% of all BC cases and is characterized by the absence of estrogen receptor (ER), progesterone receptor (PR), and human epidermal growth factor receptor 2 (HER2) expression. These receptors are typically used for targeted therapies in other BC subtypes, but in TNBC, they are absent, making therapeutic options limited. Given that no targeted therapies are available for these patients, the only systemic therapy that remains is chemotherapy. Finding new targeted therapies for TNBC is crucial to improve survival rates, reduce treatment-related side effects, and enhance quality of life for patients. To interrogate potential cancer drivers in TNBC, here we apply an optimized analytic pipeline [7] to identify 2 major arm-level SCNAs significantly associated with overall survival in TNBC patients (7p & 21q). We have pinpointed *RIPK4* on 21q as a candidate gene that underlies poor survival and demonstrated its functional importance using multiple orthogonal models. As BC remains a substantial cause of death in women [11], identifying clinically significant loci within arm-level SCNAs is of vital importance to help understand the molecular basis of metastasis, and reveal new therapeutic targets.

## Results

### Arm-level SCNAs are associated with survival of patients with TNBC

**Fig. 1:**
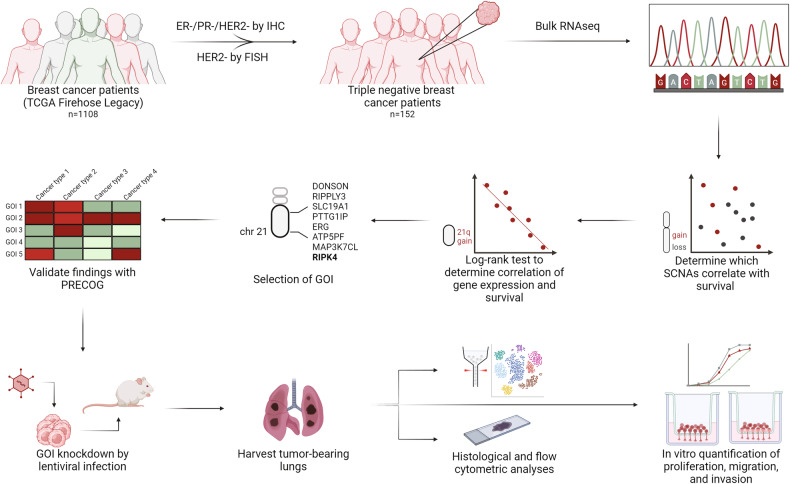
Schematic representation of the workflow employed in this study. Created with BioRender.com.

**Fig. 2:**
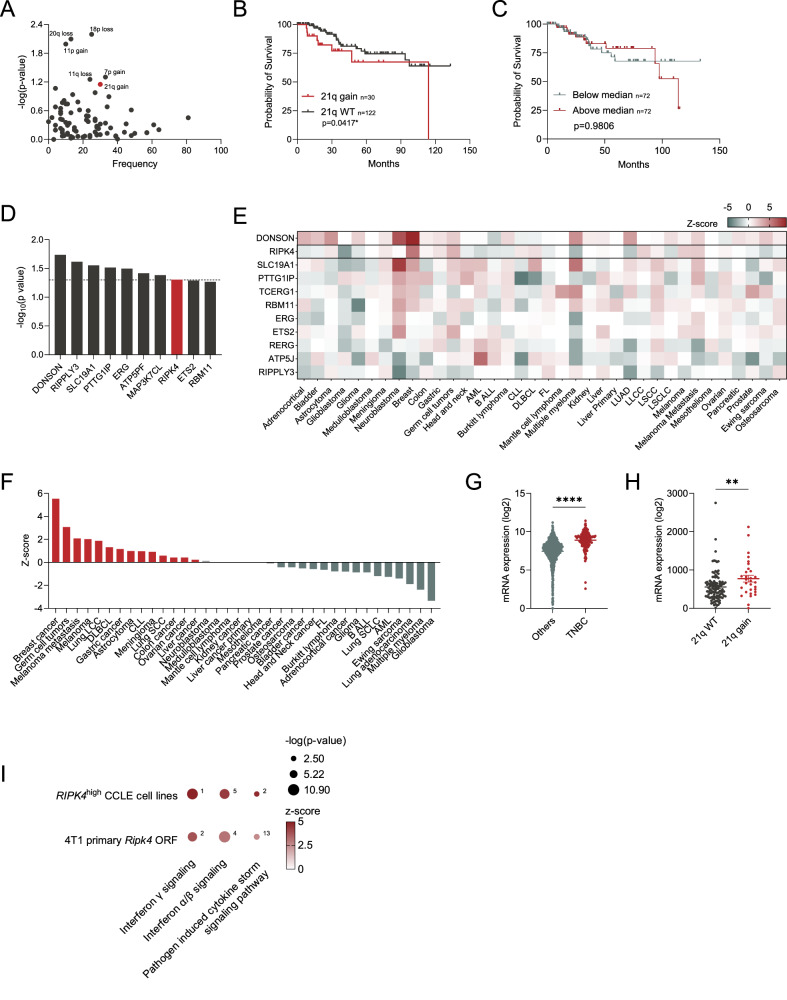
21q gain and RIPK4 expression correlate with poor survival in TNBC. **A** Average frequency and survival association of chromosome arm-level gains and losses in patients with TNBC. **B** Kaplan–Meier survival curves for TNBC patients with 21q gain versus wild-type (WT). **C** Kaplan–Meier survival curve of patients with TNBC based on the median sum of all arm-level SCNAs. **D** Genes on 21q that are most significantly correlated with survival in TNBC. **E** Survival correlation of genes on 21q across 36 cancer types from PRECOG. Red represents a negative survival correlation while green represents a positive survival correlation. **F** *Z*-scores of the association between *RIPK4* expression and survival of patients across 36 cancer types. **G** Quantification of *RIPK4* expression in TNBC tumors compared to other BC subtypes. **H** Quantification of *RIPK4* expression in tumors with a 21q gain compared to tumors that are 21q WT. **I** Selected relevant pathways from Ingenuity Pathway Analysis from primary 4T1 cell lines or human TNBC cell lines. The size of the dots represents the –log(*p* value), while the color represents the magnitude of the *z*-score. The numbers next to the dot represent the rank of the pathway when ordered based on *z*-score significance. Non-parametric Mann–Whitney *t* tests were performed for all bar graphs. *P* ≤ 0.05*, *P* ≤ 0.01**, *P* ≤ 0.001***, *P* ≤ 0.0001****.

We first assessed the association between arm-level SCNAs and overall survival of patients with TNBC to identify novel putative drivers of this aggressive form of BC. To achieve this, we curated a list of TNBC patients from the TCGA and identified 152 patients that were negative for ER/PR by immunohistochemistry (IHC), and negative for HER2 via fluorescence in situ hybridization and IHC (Fig. 1). We then performed a log-rank test to identify arm-level losses or gains significantly associated with survival. Out of all arm-level SCNAs, 21q gain and 7p gain were frequent events that significantly correlated with poor overall survival (Fig. 2A). In addition, 11q gain, 18p loss, 11q loss, and 20q loss were significantly correlated with survival, although they were much less frequent (Fig. 2A, Supplementary Table 1). Given that known oncogenes, such as *EGFR*, are located on the short arm of chromosome 7 [12–15], it was not surprising to find 7p gain as the most frequent SCNA that significantly associated with poor overall survival in our patient cohort (Fig. 2A, Supplementary Table S1). Interestingly, we found 21q gain was significantly associated with reduced overall survival (Fig. 2B). 21q gain has been identified to be more frequent in TNBC compared to other BC subtypes, but its association with survival has not been characterized in detail [16, 17]. To assess whether the link between 21q gain and survival in TNBC was merely a result of widespread arm-level copy number alterations (akin to the effects of chromosomal instability), we divided TNBC patients into two groups based on the median value of the total arm-level SCNAs present in each tumor. We then performed a Kaplan–Meier survival test and observed no significant differences in overall survival (Fig. 2C), suggesting that the prognostic value of 21q gain is more specific and predictive than simply the total quantity of arm-level SCNAs.

We next assessed the putative importance of specific genes located on 21q. We found that *DONSON*, *RIPPLY3*, *SLC19A1*, *PTTG1GIP*, *ERG*, *ATP5PF*, *MAP3K7CL*, and *RIPK4* were the only genes located on 21q whose elevated expression was significantly correlated with overall survival (Fig. 2D). *DONSON* was the most significantly correlated with survival, a gene well documented to promote BC progression [18]. To further investigate the survival association of these genes, we used the PREdiction of Clinical Outcomes from Genomic Profiles (PRECOG), an integrated cancer gene expression and clinical outcome dataset encompassing 166 independent cancer expression datasets for approximately 18,000 patients diagnosed with 39 distinct malignancies [19]. The most intriguing gene identified through this analysis was *RIPK4* which, to our knowledge, has not previously been linked to BC progression (Fig. 2E). Indeed, it was also most significantly correlated with poor survival outcome in BC over any of the other 38 cancer types (*P* = 0.000000003 in BC; Fig. 2F). Further, we also found that *RIPK4* expression was significantly higher in TNBC tumors compared to other subtypes (Fig. 2G) and that TNBC tumors harboring a 21q gain express *RIPK4* at higher levels than tumors that are 21q WT (Fig. 2H).

*RIPK4* encodes receptor-interacting protein kinase 4 enzyme (RIPK4) and is part of a family of 4 genes, *RIPK1-4*. RIPK1 and RIPK3 function as regulators of necroptosis [20], while RIPK2, most structurally similar to RIPK4, is involved in the NOD2 activation pathway, a key part of innate immune signaling [21]. RIPK4 has not been studied in the context of TNBC, but it has been shown to promote metastatic behavior of cells through epithelial to mesenchymal transition in a cell-intrinsic manner and can activate the NF-κB pathway [22–24].

To assess how *RIPK4* expression may affect tumor cells, we leveraged the publicly available RNA sequencing data from the Cancer Cell Line Encyclopedia [25] and divided 47 breast cancer cell lines based on *RIPK4* expression (Supplementary Fig. 1A, Supplementary Table 2). The top pathways predicted to be activated in *RIPK4*^high^ cell lines were interferon alpha/beta signaling, pathogen-induced cytokine storm signaling, and interferon-gamma signaling. This is consistent with the literature demonstrating that RIPK4 is known to influence cytokine production and modulate interferon regulatory factor 6 (IRF6) [26, 27]. Importantly, we observed these pathways were among the top activated pathways when we performed RNA-sequencing on 4T1-primary cells, a syngeneic murine TNBC cell line in which we overexpressed *Ripk4 by* stably infecting these cells with a *Ripk4* open reading frame (ORF- Fig. 2I, Supplementary Fig. 1B).

To explore which cells specifically express *RIPK4* within the tumor microenvironment, we leveraged single-cell RNA sequencing data from the Human Protein Atlas (HPA) and found that the expression of *RIPK4* was almost exclusively limited to breast glandular cells within normal breast tissue, with almost no expression in leukocytes [28] (Supplementary Fig. 1C, D). In contrast, other genes we discovered that correlate with poor survival, such as *DONSON*, were highly expressed by both breast tissue and infiltrating immune cells (Supplementary Fig. 1E), suggesting that *DONSON* expression assessed using bulk RNAseq (as in the TCGA) may be confounded by changes in the proportion of tumor-infiltrating cells rather than genomic changes within the tumor cells themselves. Taken together, these data implicate RIPK4 as a putative driver of TNBC progression through cancer cell-intrinsic expression of *RIPK4*.

### *Ripk4* knockdown drives aggressive TNBC phenotypes in vitro

**Fig. 3:**
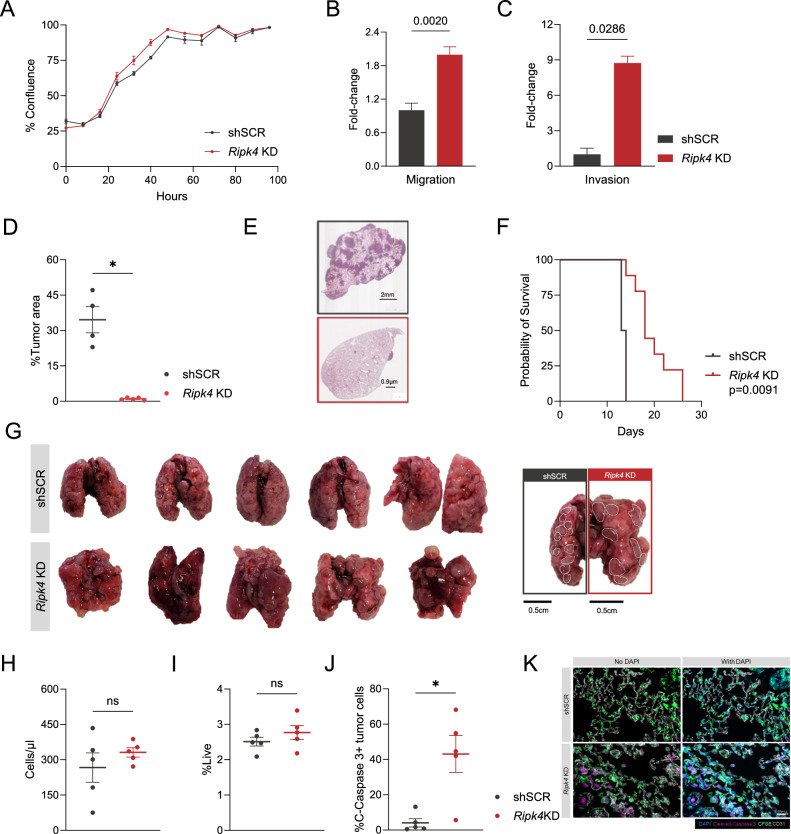
*Ripk4* KD decreases lung metastases in vivo. **A** Growth kinetics of *Ripk4* knockdown cells in vitro. **B**, **C** Bar plots illustrating the migration and invasion potential of cells with *Ripk4* knockdown in vitro. Graphs show fold-change relative to the average of controls. **D** Percent tumor area of lungs from mice injected intravenously (i.v.) with either *Ripk4* KD cells or the non-target control (shSCR) 14 days post-injection. **E** Representative images of lungs harvested and stained with H&E from mice in (**D**). **F** Kaplan–Meier survival curves of mice injected i.v. **G** Left panel: Gross anatomy of the lungs of mice injected i.v. with engineered 4T1-LuM lines at endpoint. Right panel: Dotted lines encircle tumors from representative images of lungs in the left panel. **H** Number of CFSE+ cells per µl of sample acquired able to extravasate into the lung parenchyma within 48 h. **I** CFSE+ cells able to extravasate to the lung parenchyma within 48 h as a percentage of live cells. **J** Percentage of CFSE+ tumor cells positive for cleaved caspase-3. **K** Representative images used for the quantification of cleaved caspase-3+ cells. Non-parametric Mann–Whitney *t* tests were performed for all bar graphs, except for (**B**) where a parametric *t* test was performed. *P* ≤ 0.05*, *P* ≤ 0.01**, *P* ≤ 0.001***, *P* ≤ 0.0001****.

Since mortality of BC patients is linked to metastatic disease and the lungs represent a common metastatic site, we focused our attention on the role of *RIPK4* on TNBC dissemination. We introduced a small-hairpin RNA (shRNA) against *Ripk4* by lentiviral transduction in 4T1-LuM, a murine lung-metastatic TNBC cell line that is syngeneic in immunocompetent Balb/c mice. Real-time quantitative PCR confirmed that *Ripk4* expression was reduced by 87% relative to scrambled control (shSCR – Supplementary Fig. 2A). We then explored the mechanisms by which RIPK4 promotes TNBC metastasis by investigating its contributions to specific metastatic phenotypes. We performed a series of in vitro assays aimed at determining changes in proliferation, migration, and invasion of TNBC cells with *Ripk4* perturbation. We found *Ripk4* knockdown (KD) did not result in significant changes in the proliferation (Fig. 3A). Surprisingly, *Ripk4* KD enhanced migration and invasion in vitro using transwell assays (Fig. 3B, C; Supplementary Fig. 2B, C). To gain further mechanistic understanding into the effect of *Ripk4* KD in our model, we first imaged these cells using holotomography, which revealed no observable changes in cell morphology following genetic perturbation (Supplementary Fig. 2D). Given these surprising results, we wanted to examine the genetic effect of *Ripk4* KD in these cells by performing RNA sequencing followed by Ingenuity Pathway Analysis. PAK Signaling and Rho GTPase Cycle, which are involved in the regulation of a wide variety of cell processes, such as cell survival and adhesion, were top pathways predicted to be upregulated in *Ripk4* KD cells compared to control [29, 30]. Consistent with our previous pathways analysis (Fig. 2I), interferon signaling was among the top pathways predicted to be downregulated with *Ripk4* KD (Supplementary Fig. 2E). These findings suggest that the functions of RIPK4 are likely dependent on the structural and compositional complexity of a three-dimensional tumor immune microenvironment.

### Ripk4 promotes lung metastasis of murine TNBC

To assess the metastatic capabilities of 4T1-LuM cells upon *Ripk4* KD, we performed intravenous injection into WT Balb/c mice and assessed experimental lung metastasis after 14 days. Histological analysis revealed that mice injected with *Ripk4* KD cells had significantly fewer lung metastases compared to those injected with shSCR cells (Fig. 3D, E). We then performed a survival experiment to measure the protective effect of *Ripk4* KD. We found that mice injected intravenously with *Ripk*4 KD cells had a significant survival advantage over mice injected with the control cells (Fig. 3F). However, despite the noticeable reduction in tumor burden at day 14 in mice with *Ripk4* KD, the tumors that persisted continued to grow, leading to the mice eventually succumbing to the tumor burden in their lungs (Fig. 3G). These data functionally confirm our computational predictions linking *RIPK4* with survival in patients, by demonstrating that *Ripk4* expression within tumor cells is sufficient to promote TNBC metastatic seeding and outgrowth in preclinical models.

### *Ripk4* increases survival of cancer cells at distant metastatic sites

To gain deeper insights into how *Ripk4* expression correlates with poor survival in vivo, we explored whether the expression of *Ripk4* in cancer cells might facilitate extravasation to metastatic sites, such as the lungs. Intravenously injected CFSE-stained cancer cells were given 48 h to seed the lungs before harvesting. Flow cytometric analyses revealed no discernible difference in the number of cells capable of seeding the lungs (Fig. 3H, I). Considering the well-established role of the RIPK-family protein in cell survival, we investigated whether the observed outgrowth phenotype in the *Ripk4* KD (Fig. 3D, E) group could be linked to increased cell death. Immunofluorescent analysis on the harvested lungs revealed a significant elevation in cleaved-caspase 3+ cancer cells in mice injected with the KD cells compared to controls (Fig. 3J, K). Using STRING v.12.0 [31], a database of known and predicted protein-protein interactions, we determined that RIPK4 is likely to bind to Protein kinase C delta type regulatory subunit (PRKCD). PRKCD is a serine/threonine-protein kinase that regulates apoptosis triggered by cytokine receptors [32]. These findings suggest that RIPK4 may be involved in sustaining cancer cell survival at distant metastatic sites. Moreover, examination of lungs from mice at endpoint revealed distinct macroscopic growth patterns aligning with differences in cancer cell survival (Fig. 3G). Notably, *Ripk4* KD-injected mice exhibited fewer but larger tumors compared to the numerous smaller tumors found in the lungs of mice injected with control cells. This disparity further explains the survival advantage observed in mice injected with *Ripk4* KD cells (Fig. 3C). The diminished number of surviving cells in the lung tissue takes more time to develop into expansive and dispersed tumors capable of causing mortality, in contrast to the control cells that, despite surviving extravasation, form smaller tumors covering the entire lungs at the time of death. In essence, the distinct tumor growth patterns shed light on the survival advantage conferred by *Ripk4* knockdown and underscores that RIPK4 may be mediating the rate-limiting step of cancer metastasis, seeding at the secondary site.

### The tumor immune landscape of metastatic lesions is modulated by *Ripk4*

**Fig. 4:**
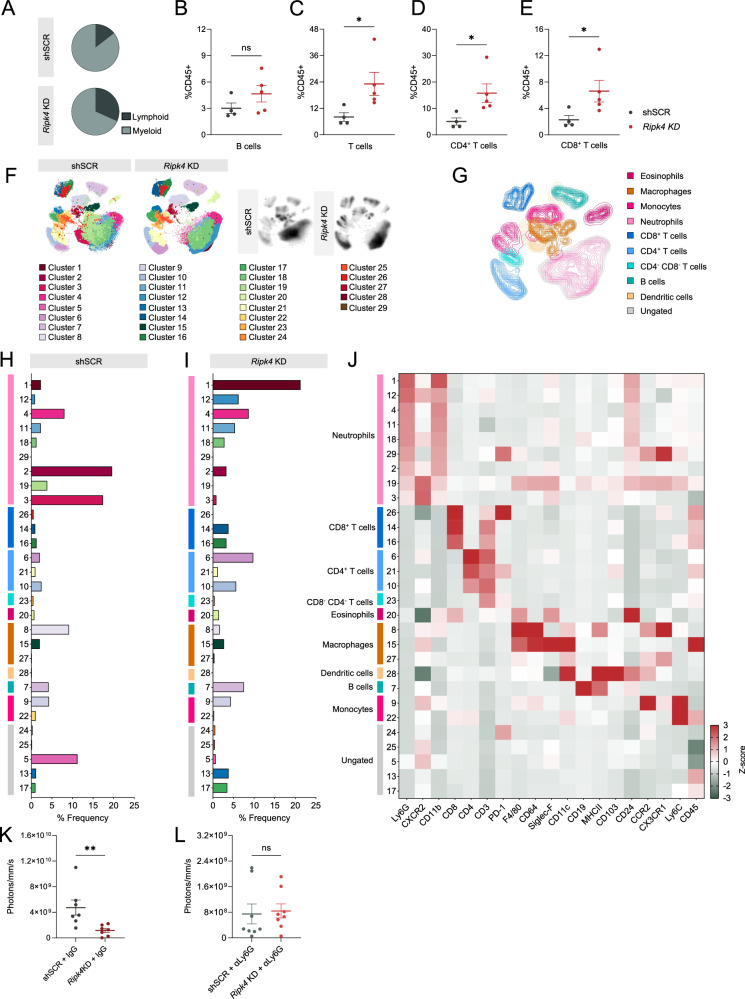
*Ripk4* expression skews the tumor immune microenvironment towards a pro-tumorigenic state. **A** Abundance of the myeloid and lymphoid compartments within the tumor immune microenvironment of mice injected i.v. **B**–**E** Relative abundance of B cells, T cells, CD4^+^ T cells, and CD8^+^ T cells, respectively, as a percentage of CD45^+^ cells. **F** UMAP depicting the 29 clusters obtained through the unsupervised clustering algorithm PhenoGraph. On the left is the non-target control and on the right is *Ripk4* KD. Density plots of the UMAPs from each group can be found on the right. **G** UMAP depicting the supervised clustering of immune cell populations present within the tumor immune microenvironment of tumor-bearing lungs. Percent frequencies of the 29 clusters in the non-target control group (**H**) and *Ripk4* KD group (**I**). **J** Heatmap representing the relative expression level of each marker across each cluster. Color boxes along the left *y*-axis match the supervised clustering cell type assignment. **K** Bioluminescence signals from mice injected i.v. with 4T1-LuM cells and treated with IgG, 13 days post-injection. **L** Bioluminescence signals from mice injected i.v. with 4T1-LuM cells and treated with anti-Ly6G, 13 days post-injection. Non-parametric Mann–Whitney *t* tests were performed for all bar graphs. *P* ≤ 0.05*, *P* ≤ 0.01**, *P* ≤ 0.001***, *P* ≤ 0.0001****.

Given that RIPK4 has been shown to play a role in the activation of the NF-κB pathway, and its expression negatively correlates with the predicted presence of T cells in human ovarian cancer [22, 33], we reasoned that RIPK4 may be modulating the tumor microenvironment. To comprehensively assess how RIPK4 shapes the immune landscape of TNBC, we performed spectral flow cytometry on our experimental lung metastasis assay using a 19-plex antibody panel. We first performed a broad characterization of major immune cell types and found a significant increase in lymphoid cells and a relative decrease in myeloid cells in lungs harboring *Ripk4* KD metastases compared to shSCR controls (Fig. 4A; Supplementary Fig. 3A, B). We performed these assays 14 days post injection, when both groups had tumors in the lungs but with clearly distinct growth patterns (Fig. 3E). Within the total leukocyte pool, this increase in lymphocytes following *Ripk4* KD was driven by an increase in CD4^+^ and CD8^+^ T cells, with no change observed in B cells (Fig. 4B–E). We also found a significant decrease in the percentage of eosinophils and non-tissue resident macrophages following *Ripk4* KD compared to controls, while tissue-resident macrophages remained at similar levels between both groups (Supplementary Fig. 3C–E). Given that a hallmark feature of TNBC is an “immune-cold” microenvironment where lymphocyte infiltration into the tumor is limited [15, 34, 35], our data indicate that targeting *RIPK4* may foster an increase abundance of lymphocytes at the tumor site. To further dissect specific populations within the microenvironment that were sensitive to *Ripk4* alterations, we performed unsupervised clustering of our spectral flow cytometry data via PhenoGraph [36]. Partitioning high-dimensional data into clusters using this approach enables the identification of rare phenotypic subsets or functional states, thus revealing potential biomarkers or novel cell populations that are more likely to be overlooked using traditional gating approaches. Using PhenoGraph, we identified 29 clusters in total (Fig. 4F, H–J), including 9 neutrophil clusters (1, 12, 4, 11, 18, 29, 2, 9, 3), 3 CD8^+^ T cell clusters (26, 14, 16), 3 CD4^+^ T cell clusters (6, 21, 10), and 5 monocyte/macrophage clusters (9, 22/8, 15, 27). We also saw individual clusters for CD4^−^CD8^−^ double-negative T cells (23), eosinophils (20), dendritic cells (28), and B cells (7), suggesting reduced functional heterogeneity compared to other major cell types; as well as 5 leukocyte clusters that were undefined by the markers in our panel.

Using this dataset, we quantified the frequencies of individual clusters. Consistent with our supervised gating results (Fig. 4D, E, G), we found increased frequencies of CD4^+^ and CD8^+^ T cell clusters in the *Ripk4* KD group (Fig. 4H, I). Moreover, monocyte-derived macrophages (CD11b^+^ CD64^+^ F4/80^+^ CCR2^+^) were increased in the control group, while tissue-resident macrophages (CD11b^-^ F4/80^+^ CD64^+^ Siglec-F^+^ CD11c^hi^) remained unchanged (Fig. 4H–J). Interestingly, while neutrophils represented the largest leukocyte population in metastases (Fig. 4G–I), we did not see a significant difference in the frequency of total neutrophils between control and *Ripk4* KD tumors (Supplementary Fig. 3F). Instead, the most striking changes were found amongst neutrophil clusters, where clusters 1, 12, 11, and 18 were increased in the *Ripk4* KD metastases compared to shSCR control, and clusters 2, 19, and 3 were reduced (Fig. 4H, I). The most abundant neutrophil cluster in *Ripk4* KD metastases (and the most abundant of *all* immune clusters) had highest levels of Ly6G and intermediate levels of both CXCR2 and Siglec-F (Fig. 4H–J), coinciding with reduced metastatic burden. Given that high Siglec-F in neutrophils imparts a pro-tumorigenic phenotype [37], the reduced expression of Siglec-F aligns with our observation of reduced metastatic burden in response to *Ripk4* KD.

Neutrophils are highly heterogeneous in their ability to elicit either pro- or anti-tumorigenic effects on cancer metastasis [38]. Given the impact of RIPK4 on metastasis in our model, we further explored the functional link between tumor cell-RIPK4 and neutrophils. First, we explored whether RIPK4 regulates CXCR2 ligands using a cytokine array on tumor cell-conditioned media. Among the top proteins that were differentially produced, we found CXCL1 production by *Ripk4* KD cells was upregulated compared to shSCR control cells (Supplementary Fig. 3G, H), potentially acting as a chemotactic factor. Next, we asked whether the efficacy of *Ripk4* KD was dependent on neutrophils. To achieve this, neutrophils were depleted in our experimental lung metastasis assay using antibodies targeting Ly6G [39]. We found that the ability of *Ripk4* KD to reduce metastasis was only observed in neutrophil-proficient IgG-treated mice (Fig. 4K, Supplementary Fig. 3I) and was lost in neutrophil-deficient mice treated with anti-Ly6G (Fig. 4L, Supplementary Fig. 3I). These findings indicate that the phenotype we observed with *Ripk4* perturbation, is in part mediated through interaction with neutrophils within the tumor immune microenvironment.

## Discussion

In this study, we investigated the association between arm-level SCNAs and overall survival in TNBC. Using data from The Cancer Genome Atlas we identified frequent SCNAs, such as 21q gain and 7p gain, that significantly correlated with poor overall survival. Further, we identified several genes located within the 21q arm, including *RIPK4*, whose elevated expression was significantly associated with poor survival. Meta-analysis confirmed *RIPK4* as a significantly correlated gene with poor survival in breast cancer. We also demonstrated that *Ripk4* promotes lung metastasis in a murine TNBC model. Reduced expression of *Ripk4* skewed the tumor immune microenvironment, marked by elevated T cells, and altered neutrophil subsets. Importantly, the effect of RIPK4 on lung metastasis was in part dependent on the presence of neutrophils, as *Ripk4* KD had no effect in neutrophil-deficient models. Further, our in vivo experiments demonstrated that while *Ripk4* knockdown resulted in a noticeable reduction in tumor burden at early time points, the surviving cancer cells exhibited distinct growth patterns. This observation indicates that RIPK4 may function as a key mediator of the rate-limiting step in cancer metastasis – the seeding and survival at distant sites. The altered growth patterns, with fewer but larger tumors in the *Ripk4* knockdown group, underscore the potential of targeting RIPK4 to impede the progression of metastatic lesions.

DNA CNVs are an important component of genetic diversity [40]. While characterizing germline CNVs has provided insight into their role in the susceptibility to a wide range of diseases, somatic CNVs (often referred to as SCNAs) can hold valuable information for identifying regions of the genome involved in disease phenotypes such as cancer [41]. SCNAs can both activate oncogenes and inactivate tumor suppressor genes; as such, the major challenge is how to identify potential ‘driver’ events (i.e., responsible for a given phenotype) versus ‘passengers’ events (i.e., acquired during cellular evolution but do not contribute towards a particular phenotype) [42]. This is particularly difficult for arm-level SCNAs, since broad amplification or deletion of nearly entire chromosome arms can contain hundreds of genes that each can either be driver or passenger events [43]. This study underscores the complexity of distinguishing driver events from passenger events within arm-level SCNAs. While frequency is acknowledged as a valuable metric, it is essential to recognize that not all drivers are necessarily associated with poor prognosis. Some drivers may exhibit a neutral or even favorable prognosis, adding a layer of complexity to their clinical implications. Distinguishing driver genes from passenger genes within arm-level SCNAs has remained a challenging but important problem in cancer genetics. Additional considerations further complicate this problem: First, SCNAs do not necessarily correlate with concomitant changes in gene/protein expression, despite the amplification or heterozygous deletion of a particular locus [7]. Second, most studies that attempt to identify arm-level SCNAs that are important in cancer simply look at the frequency of an event, and assume that if something happens often, it must be significant. However, frequent genetic events do not necessarily contribute to a given phenotype. A classic example of this is the *TTN* gene, one of the most frequently mutated genes across cancers simply due to its size, that does *not* contribute to oncogenesis [44]. Third, in BC, certain SCNAs may be vital for tumor initiation, but not progression or metastasis [45, 46]. As most BC patients do not die from primary tumors, these SCNAs are of less immediate therapeutic importance. This study highlights the significance of arm-level somatic copy number alterations in triple-negative breast cancer and identifies *RIPK4* as a potential driver gene associated with poor overall survival and metastasis. The findings underscore the complexity of distinguishing driver events from passenger events within arm-level SCNAs and emphasize the importance of considering gene expression and functional effects. One limitation of our study is the absence of validation through human TNBC cell lines. Despite this, the significant role of RIPK4 in modulating the tumor immune microenvironment is well established in our findings, therefore, we chose not to pursue validation using immunocompromised models. Understanding the role of RIPK4 and its impact on the tumor immune microenvironment may provide insights into TNBC progression and offer potential therapeutic targets for this aggressive form of breast cancer.

## Materials and methods

### Clinical data

Clinical data from the TCGA (bulk RNAseq on tumors) and the CCLE (bulk RNAseq on TNBC cell lines) were accessed through the online platform cBioPortal (accessed September 2022).

### Mice

Female Balb/c mice were obtained from Jackson Laboratory and housed in pathogen-free conditions. A minimum of one week was given for acclimatization before any experimental procedures were conducted. Mice were used for experiments between 5 to 7 weeks of age and sacrificed in the morning at the defined experimental endpoint. All protocols were reviewed and approved by McGill University Animal Care Committee and conformed to standards by Canadian Council on Animal Care.

### Cell lines

The 4T1 LuM cell line was generously gifted from Dr. Peter Siegel (McGill University) [47]. All cell lines were confirmed to be mycoplasma-free. 4T1 LuM cells were maintained in RMPI 1640 media supplemented with 10% FBS and 1% penicillin/streptomycin. Puromycin selection for genetically engineered cell lines containing shRNAs was maintained at a concentration of 3.5 ug/ml (Sigma). HEK293T Lenti-X cells were used for lentivirus transductions. HEK293T cells were cultured in DMEM supplemented with 10% FBS and 1% penicillin/streptomycin. 4T1 primary cells were obtained from ATCC and cultured in RPMI 1640 media supplemented with 10% FBS and 1% penicillin/streptomycin.

### Genetic perturbation

HEK293T Lenti-X cells were transfected with plasmids using Lipofectamine 2000 Transfection Reagent (Thermo Fisher) and following the manufacturer’s instructions. Transductions were carried in 6-well dish and 1 μg of shRNA plasmids (pLKO.1) or 1 μg of ORF (EX-Mm10838-Lv151-GS; Genecopoeia) or empty control (pReceiver-Lv151; Genecopoeia) were added with 1 μg of psPAX2 packaging vector and 0.5 μg of pMD2.G envelope vector. Viral supernatant was collected after 24 h following transfection and was added to target cells along with 1 ml of fresh complete media and polybrene at a final concentration of 8 ug/ml (Sigma Aldrich). A second round of infection was performed 24 h later. Target cells were selected with 3.5 μg/ml puromycin (Sigma Aldrich) or 300 μg/mL of G418 (Wisent) for a minimum of 7 days before confirming knockdown or overexpression. All shRNA constructs were obtained from the High Throughput Screening Facility (Life Science Complex, McGill University). For bioluminescence imaging, super-infections were repeated as described using the pHIV-Luc-ZsGreen (Addgene). Three days before injections, cells were FACS-sorted based on green fluorescent protein expression and only positive cells were injected in the mice.

### RT-qPCR and western blot for gene knockdown confirmations

Qiagen RNAeasy Kits were used to extract RNA from target cells following the manufacturer’s protocol. RNA purity was verified using the DeNOVIX DS-11 FX pectrophotometer/Fluorometer. cDNA was synthesized overnight, and the reaction was carried following recommendation from supplier (Applied Biosystems). qRT-PCR was performed using PowerUP SYBR Green Master Mix (Appliedbiosystems) along with the relevant primers (IDT) on a QuantStudio6Flex.

Primers:

*Actinb*: RV: 5’CTGGATGGCTACGTACATGG 3”; FW: 5’ACCTTCTACAATGAGCTGCG 3′.

*Ripk4*: RV: 5’CACATCAGCCTTCTCCTCTATG 3′; FW: 5’CTTTGACCTCAGAGGGCTATAC 3′.

### Transwell assays

For the migration assay, 4T1 LuM cells were seeded into the top chamber of a Transwell (8μm pores, 24-chamber format, Corning) at a density of 100,000 cells in 250 μl of serum-free RPMI. 750 μl of conditioned media from WT 4T1 LuM cells was added to the lower chamber of the well as chemoattractant. For the invasion assay, Transwells were coated with 10 μg of Matrigel (Corning) diluted in 100 μl of serum-free media and left to polymerize at room temperature overnight. Then, 100,000 cells in 150 μl of serum-free media were seeded onto the Matrigel layer. For both assays, cells were allowed to migrate for 24 h. The wells were then rinsed in PBS before being fixed in 4% paraformaldehyde (PFA) and stained with 1:5000 DAPI in PBS. The transwell membranes were mounted on a glass microscope slide using Dako Mounting Medium. The slides were imaged using the EVOS 5000 cell imaging system (ThermoFisher; 5 images per membrane, 4 membranes per slide) and the cell counts were quantified using the ImageJ software. All assays were done in replicates of four.

### Proliferation assay

4T1 LuM cells were seeded in 96-well plate at a density of 5000 cells in 250 μl of media. The plate was imaged twice a day at 12 h interval using an Incucyte S3 (Satorius) for one week and percent confluency was calculated based on the confluency map.

### Experimental lung metastasis assay

5–7-weeks-old female Balb/c were injected with 500,000 4T1 LuM cells suspended in 200 μL dPBS via lateral tail vein. Mice were monitored every three days and were sacrificed at a 14-day experimental endpoint or until a mouse showed sign of respiratory distress. For survival trials, the mice were monitored daily after the 14-day endpoint and were sacrificed as debilitating symptoms arose. The lungs were collected from the mice; one lobe was fixed in formalin for 24 h and transferred in 70% ethanol for at least 24 h before being sent for histological processing, and the remaining tissue was processed for flow cytometry (described below). HALO Image analysis software (Indica labs v.2.0.9) was used for histology analysis, and metastasis burden was measured as tumor area as a percentage of total lung area. For the short-term (48 h) metastasis assays, cells were stained using CellTrace CFSE (ThermoFisher Scientific) according to the manufacturer’s recommendation. Cells were injected in the lateral tail vein of mice and allowed to seed the lungs for 48 h. At the time of harvest, a cut was made in the right atrium of the heart and 10 mL of 1X PBS was pushed through the left ventricle to flush the systemic circulation and any cells that did not enter the lung parenchyma. Following this, 10 mL of 4% paraformaldehyde (PFA) was pushed through the ventricle to allow proper fixation of the lungs and ensure proper flushing of the lung vasculature. One lung was processed for flow cytometry as described below and the other lung was collected, fixed for 24 h in 4% PFA, followed by 24 h in 20% sucrose in 1X PBS. Lungs were then embedded in O.C.T. compound (Sakura) and kept at –80 °C.

### Flow cytometry

Lungs were harvested from mice and minced using a razor blade, rinsed with FACS buffer (dPBS + 2% FBS) and filtered through a 40 μm cell strainer. Red blood cell lysis (BD) was performed for 15 min in the dark on the samples before 35% Percoll (Sigma-Aldrich) was added to resuspend the pellets. Cell suspensions were centrifuged for 15 min at 360 × *g* (no breaks) to enrich the leukocyte fraction of the samples. The cells were resuspended in dPBS containing live/dead stain and stained for 30 min at room temperature in the dark. Then, a combination of fluorochrome-conjugated antibodies at optimized concentrations in a 1:1 FACS to Brilliant stain buffer (BD) for 30 min at 4 °C. Samples were fixed with Fix/Perm solution (eBioscience) for 1 h at 4 °C. CountBright beads (ThermoFisher Scientific) were added to samples of the 48 h metastasis assay to obtain absolute count of tumor cells in the lungs. Samples were acquired within 7 days of processing on a Cytek Aurora Spectral Flow Cytometer. Flow cytometry data visualization and gating (Supplementary Fig. 4) was performed using FlowJo v10.8.1 (BD) and UMAP, Phenograph, and Cluster Explorer plugins were used (downloaded from FlowJo Exchange). The antibody panel can be found in Supplementary Table 3.

### Histology slide preparation and imaging

Harvested lungs were inflated and fixed with formalin for 24 h, then transferred to 70% ethanol for a minimum of 24 h prior to paraffin embedding. Sectioning was performed by the Histology Core Facility at the Goodman Cancer Institute and Life Sciences Complex, McGill University. Slides were stained with H&E and imaged with an Aperio ImageScope. Tumor burden was calculated using HALO Image analysis software (Indica labs v.2.0.9). The cytonuclear analysis was performed on each lung section. Holotomography on the 4T1-LuM lines was performed using a Tomocube HTX1 (Tomocube Inc.).

### Immunofluorescent histochemistry

O.C.T. Compound (Sakura) embedded lungs were cut into 5-µm-thick sections, thawed, and dehydrated at room temperature, then rehydrated in 1X PBS before the staining process. Tissues were blocked with Dako blocking reagent (1 h, room temperature; Agilent). Primary antibody (Cleaved Caspase-3; 9661L; Cell Signaling and CD31; AF3628; R&D Systems) was diluted 1:100 in Dako antibody diluent and incubated for 1 h at room temperature. Following this, tissues were rinsed in 1X PBS, incubated for 1 h at room temperature with AlexaFluor secondary antibody (1:500; Invitrogen), and subsequently rinsed in 1X PBS. DAPI (4,6-diamidina-2-phenylindole) was employed for counterstaining. Slide scanning was performed using an Axio Scan (Zeiss) and QuPath (v.0.4.0) was used for analysis.

### Cytokine array

The 0.45 um filtered culture supernatant from the 4T1-LuM cells were used with the Proteome Profiler Mouse XL Cytokine Array kit (Bio-techne) to assess for 111 cytokines. Kit was used as per manufacturer’s recommendation. The nitrocellulose membranes were exposed and imaged using the Amersham Imager 600.

### Neutrophil depletion

For the depletion of neutrophils in vivo, we used anti-Ly6G (clone 1A8) with a mouse IgG2a antibody against rat kappa immunoglobulin (clone MAR 18.5) [39], both at a dose of 4 mg/kg body weight (rat IgG2a isotype control, BioXcell). Anti-Ly6G was administered via intraperitoneal injection 2 days before tumor cell injection. Treatment was continued throughout the trial and injections were repeated every 3 days. Neutrophil depletion was validated by flow cytometry at endpoint.

### Bioluminescence imaging

Mice were injected intraperitoneally with 200 uL of luciferin solution. The luciferin solution was prepared from powdered luciferin resuspended in PBS and filtered through 0.2 μm filter, VWR, and s calibrated to 10 μL/g of body weight. At 5 min post-injection, images were acquired using the IVIS Spectrum In Vivo Imaging System (Perkin Elmer). Quantification of BLI was performed on the accompanying software, IVIS Living Image (Perkin Elmer).

### Bulk RNA sequencing

RNA extraction was done using the RNeasy Plus kit from Qiagen. Subsequent RNA sequencing and quality control procedures were conducted at the McGill University Genome Centre. A SMART-Seq library preparation kit was employed, and sequencing with 2 × 100 base pairs was executed on an Illumina HISeq 2000. Fastp (v.0.20.0) was employed to gather quality control metrics for the raw reads. For alignment to the National Center for Biotechnology Information mouse genome build 38 v.96, the STAR aligner (STAR-2.6.1b) was used, and the resulting RNA sequences were sorted by coordinates. Removal of alignment duplicates was accomplished through Sambamba (v.0.7.0). Gene quantification was carried out using featureCounts (v.2.0.0). DESeq2 (v.1.24.0) was then applied for the normalization of feature counts and the identification of differentially expressed genes. The extraction of HGNC symbols and their integration into the DESeq2 results data frame were achieved using biomaRt (v.2.40.4), utilizing the ‘musculus_gene_ensembl’ dataset from Ensembl Release 96 (April 2019). Subsequent analyses were performed with Ingenuity Pathway Analysis tool from Qiagen. *P*-value cutoffs between 0.01 and 0.001 were used to obtain a list of 1500 differentially expressed genes at most.

### Quantification and statistical analysis

Figures and heatmaps were generated in Prism 9.5.1. All graphs are presented as mean ± SEM unless indicated otherwise. Statistical analyses were performed as described in figure legends and plotted using Prism 9.5.1. Data were determined to be non-parametric as per the Shapiro–Wilk normality test, appropriate statistical test was performed accordingly and specified in the figure legend. *P*-value smaller than 0.05 were considered as significant.

### Supplementary information

Supplementary Legends

Supplementary Figure 1

Supplementary Figure 2

Supplementary Figure 3

Supplementary Figure 4

Supplementary Table 1

Supplementary Table 2

Supplementary Table 3

## Data Availability

Data are available upon reasonable request. All RNA sequencing acquired in this study will be deposited and can be accessed using the NCBI BioProject accession number PRJNA1102119.
