# Supplementary material for: Exploring the prognostic significance of arm-level copy number alterations in triple-negative breast cancer
Source: Oncogene. 2024 May 14;43(26):2015–24. doi: 10.1038/s41388-024-03051-y (PMC11196216; doi:10.1038/s41388-024-03051-y)
Supplement: Supplementary file 1 — Supplementary Legends [file 41388_2024_3051_MOESM1_ESM.docx]

**Supplementary figure legends and supplementary table legends**

**Supplementary Figure 1**

**A.** *RIPK4* expression in TNBC cell lines from the CCLE. *RIPK4*^high^ cells are defined as the top 25% expressors while the *RIPK4*^low^ cells are defined as the bottom 25% expressors. **B.** Normalized counts or *Ripk4* in 4T1 primary cells genetically engineered to express an empty vector or an ORF for *Ripk4*. **C.** Transcript per million (TPM) of *RIPK4* in cells from breast tissues measured by scRNA sequencing. **D.** TMP of *RIPK4* in immune cells measured by scRNA sequencing. **E.** TPM of *DONSON* in immune cells measured by scRNA sequencing. All data were retrieved on the Protein Atlas. Two-tailed Student’s t-test, P ≤ 0.05*, P ≤ 0.01**, P ≤ 0.001***, P ≤ 0.0001****

**Supplementary Figure 2**

**A.** Fold-change expression of *Ripk4* in 4T1-LuM cells calculated by ΔΔCt. **B.** On the top are representative images of non-target control cells and *Ripk4* KD cells that were able to migrate through a transwell stained with DAPI. On the bottom are greater magnifications of the boxed areas on the top panel. **C.** T On the top are representative images of non-target control cells and *Ripk4* KD cells that were able to migrate through a Matrigel layer across a transwell insert stained with DAPI. On the bottom are greater magnifications of the boxed areas on the top panel. **D.** Representative holotomography images of the engineered 4T1-LuM cells that shows no marked differences between the controls and the *Ripk4* KD lines. **E.** Selected relevant pathways from IPA analysis from the 4T1-LuM *Ripk4* KD compared to scrambled control. The size of dots represents the –log(p-value), while the color represents the magnitude of the z-score.

**Supplementary Figure 3**

**A.** Frequency of lymphocytes as a percentage of CD45^+^ cells. **B.** Frequency of myeloid cells as a percentage of CD45^+^ cells. **C.** Abundance of alveolar macrophages as a percentage of CD45^+^ cells. **D.** Monocyte-derived macrophages abundance as a percentage of CD45^+^ cells. **E.** Eosinophils as a percentage of CD45^+^ cells. **F.** Frequency of neutrophils as a percentage of CD45^+^ cells. **G.** Fold-change measurement of CXCL1 cytokine in cell culture supernatant of *Ripk4* KD cells relative to the shSCR cells. **H.** Images of the whole cytokine arrays used to perform the measurement. CXCL1 is boxed. **I.** Blood neutrophil quantification at endpoint as a percentage of CD45+ cells. Neutrophils were defined as CD45+ CD11b+ Ly6G+ Ly6Cmid. Non-parametric Mann-Whitney t-tests were performed for all bar graphs, but **I** (One-way ANOVA followed by Sidak’s multiple comparisons). P ≤ 0.05*, P ≤ 0.01**, P ≤ 0.001***, P ≤ 0.0001****.

**Supplementary Figure 4**

Gating strategy for flow cytometry.

**Supplementary Table 1**

Frequency of arm-level copy number alteration and associated log-rank p-value.

**Supplementary Table 2**

RPKM value of *RIPK4* transcript in triple negative breast cancer cells included in the CCLE.

**Supplementary Table 3**

Antibody information used for flow cytometry.
