# Supplementary figures and images for: Exploring the prognostic significance of arm-level copy number alterations in triple-negative breast cancer
Source: Oncogene. 2024 May 14;43(26):2015–24. doi: 10.1038/s41388-024-03051-y (PMC11196216; doi:10.1038/s41388-024-03051-y)

A

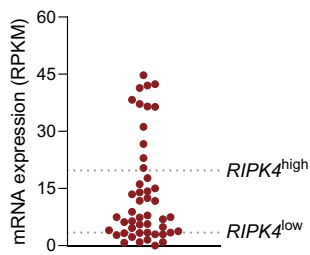

B

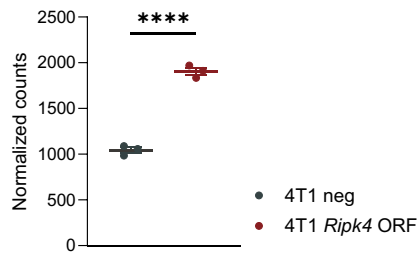

C

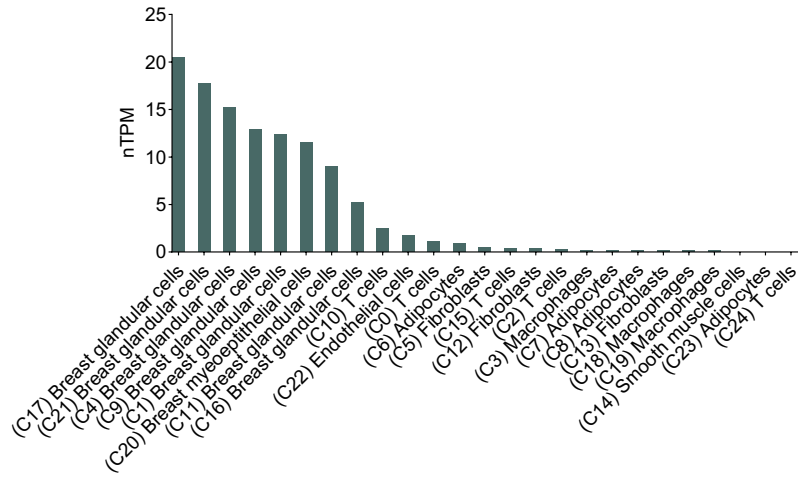

D

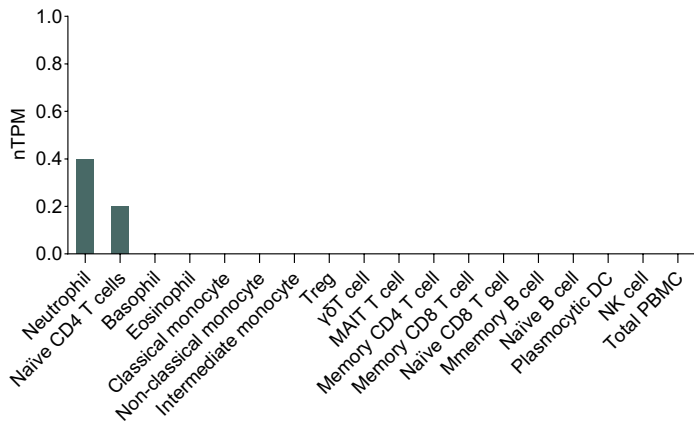

E

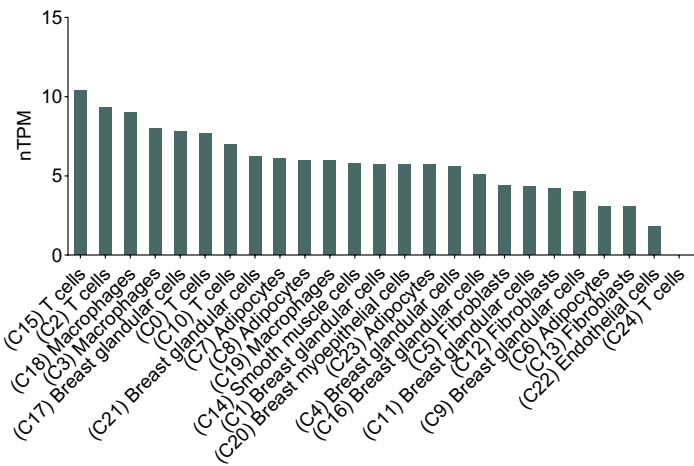

Supplement: Supplementary file 2 — Supplementary Figure 1 [file 41388_2024_3051_MOESM2_ESM.pdf]

A

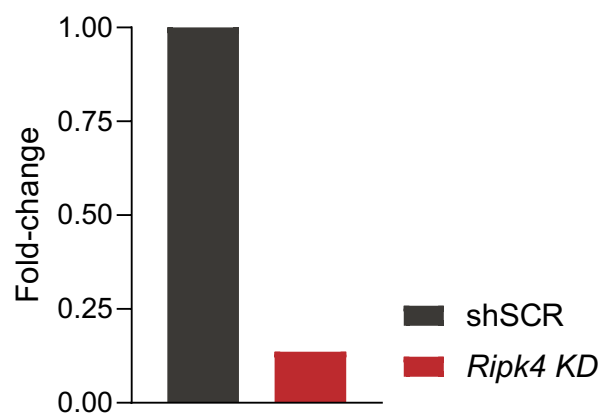

B

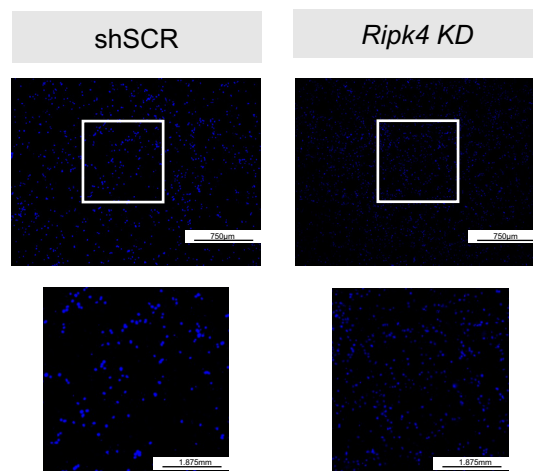

C

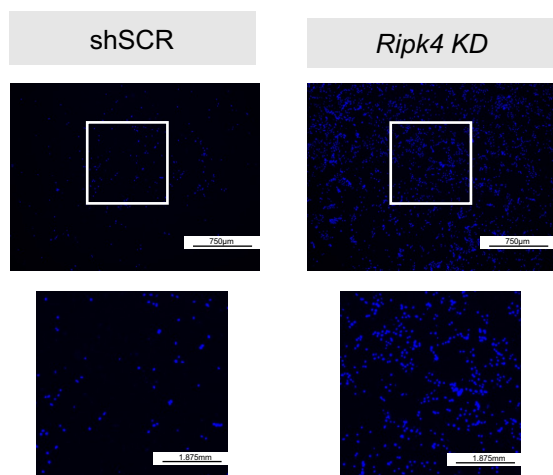

D

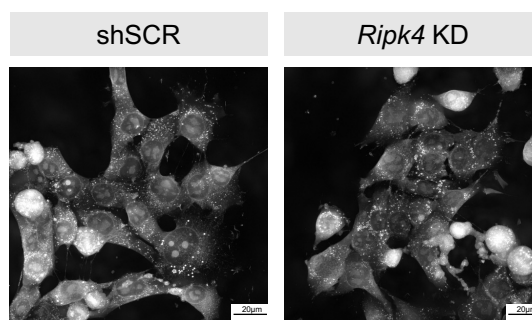

E

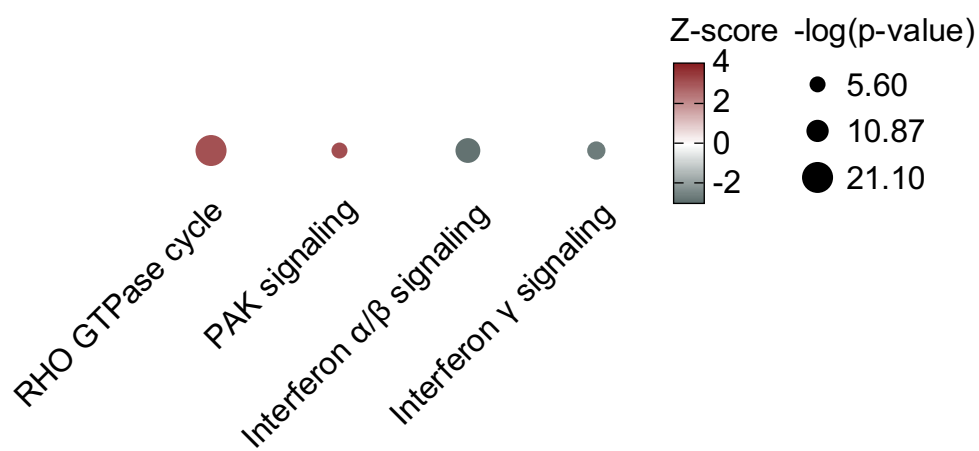

Supplement: Supplementary file 3 — Supplementary Figure 2 [file 41388_2024_3051_MOESM3_ESM.pdf]

**A**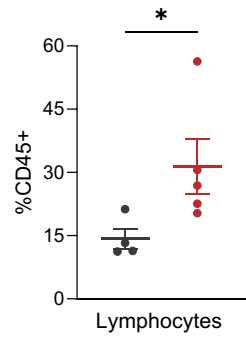**B**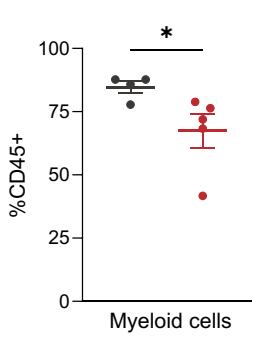**C**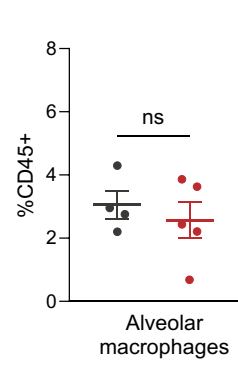**D**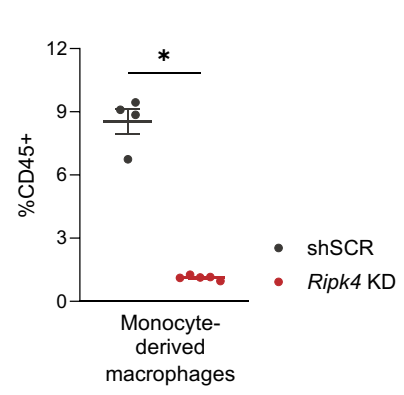**E**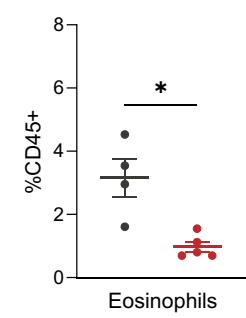**F**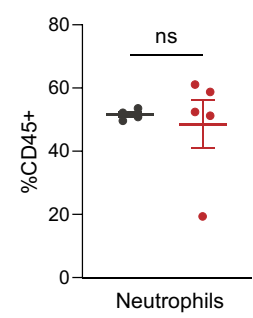**G**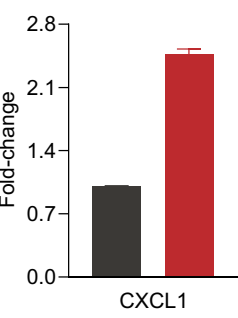**H**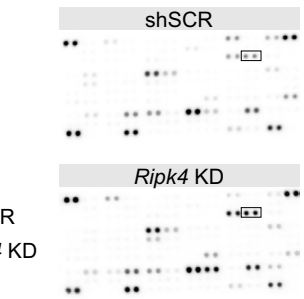**I**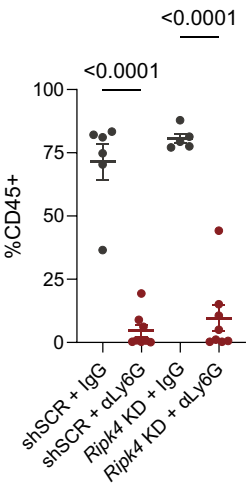

Supplement: Supplementary file 4 — Supplementary Figure 3 [file 41388_2024_3051_MOESM4_ESM.pdf]

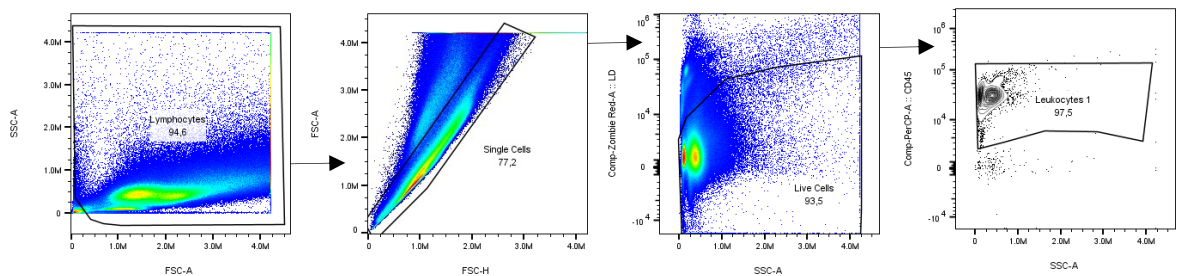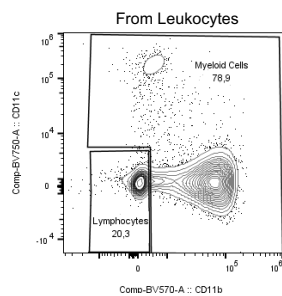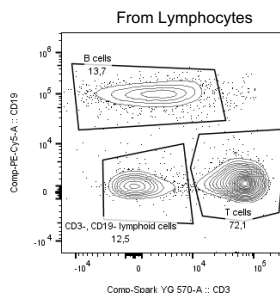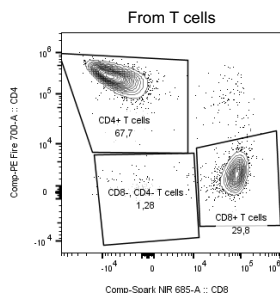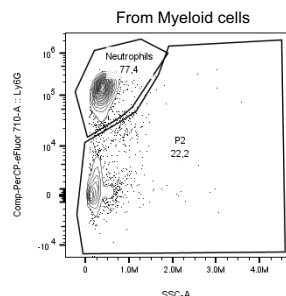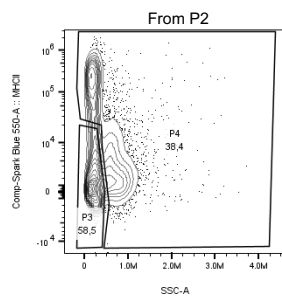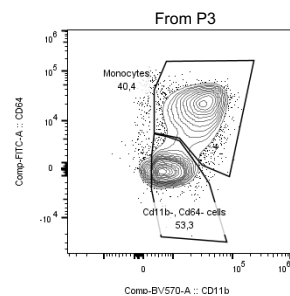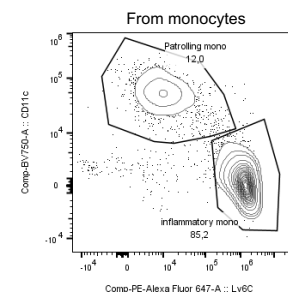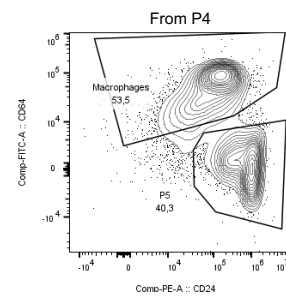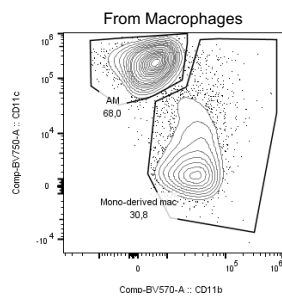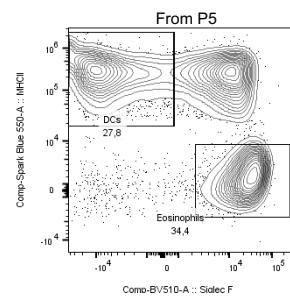

Supplement: Supplementary file 5 — Supplementary Figure 4 [file 41388_2024_3051_MOESM5_ESM.pdf]
